# Innovative Wearable Platform for Synchronized Biosignals Acquisition: A Proof of Concept in a Cuff-Less Blood Pressure Monitoring Case Study

**DOI:** 10.1109/JTEHM.2026.3687981

**Published:** 2026-04-27

**Authors:** Alessio Serrani, Andrea Aliverti

**Affiliations:** Dipartimento di ElettronicaInformazione e BioingegneriaPolitecnico di Milano Milan 20133 Italy; Kalpa S.r.l. Milan 20099 Italy

**Keywords:** Cardiovascular dynamics, cuff-less blood pressure monitoring, pulse arrival time, wireless wearable platform.Clinical Impact—The proposed fully wireless platform can support clinicians in tracking cardiovascular dynamics outside clinical environments and helps patients undergo unobtrusive monitoring during daily life, improving comfort and long-term adherence

## Abstract

Objective: This study evaluates the performance of a fully wireless multi-node wearable platform equipped with a sub-microsecond synchronization engine in a clinically relevant scenario. The prototype system is used in a cuff-less blood pressure monitoring application, based on pulse arrival time derived from synchronized ECG and PPG signals. Methods: The system integrates a custom 2.4 GHz synchronization protocol and Bluetooth Low Energy for data transmission. Nineteen healthy subjects completed a treadmill protocol designed to induce transient hemodynamic perturbations representative of daily-life physical activities. PAT values extracted from ECG and PPG signals acquired before and after exercise were compared with reference systolic blood pressure (SBP) measurements, intermittently sampled using a validated oscillometric device. The experimental protocol and analysis were designed to reflect realistic home monitoring scenarios, including limited user interaction during measurements. Results: Immediately after exercise, significant deviations from baseline were observed in computed PAT ($p< 10^{-18}$) and SBP ($p< 10^{-7}$). PAT recovery trend was accurately modeled by a mono-exponential function ($R^{2}=0.98$). Recovery indices derived from PAT and SBP were strongly correlated (Pearson’s $r=0.93$, $p< 0.001$) with high concordance ($\rho _{c}=0.885$), although Bland-Altman analysis revealed subject-specific variability (LoA: -34.36% / 37.53%). Conclusion: The proposed platform enables continuous synchronized multi-signal acquisition for extracting PAT dynamics and tracking blood pressure fluctuations under realistic home-monitoring conditions, with minimal additional user interaction. These results support the operational feasibility of wirelessly synchronized architectures for cardiovascular monitoring in daily-life scenarios, promoting integration into remote health assessment workflows beyond traditional intermittent cuff-based measurements.

## I. Introduction

Recent developments in wearable technology and electronics miniaturization have accelerated the diffusion of Body Sensor Networks (BSNs), distributed systems of autonomous sensing nodes cooperating to collect and transmit biosignals in real time. These platforms enable continuous physiological monitoring for clinical and fitness applications and offer new opportunities for longitudinal supervision of chronic and subclinical conditions outside traditional healthcare facilities. However, most conventional systems still rely on wired connections to ensure inter-sensor synchronization, compromising comfort and wearability and limiting their suitability for prolonged use during daily activities. Indeed, although wired configurations guarantee precise temporal alignment through a shared physical clock, they limit user movements and may reduce patient adherence in real-world monitoring scenarios.

To overcome these limitations, we previously proposed and technologically validated a fully wireless, multi-node platform achieving sub-microsecond synchronization accuracy [1], [2], [3]. The architecture is based on a hierarchical design with distributed sensing modules, each adaptable to different analog transducers, and a central gateway for data aggregation. Inter-node synchronization is achieved via a proprietary low-level 2.4 GHz protocol minimizing jitter and overhead, while Bluetooth Low Energy (BLE) ensures data transmission toward the external gateway.

The key innovation of the proposed system lies in this dual-protocol wireless architecture, in which a lightweight proprietary synchronization layer is specifically optimized for precise timing alignment, while BLE is reserved for standard-compliant data transmission. This separation enables distributed wearable sensing nodes to remain synchronized without sacrificing communication interoperability, thereby creating an engineering infrastructure that can be reconfigured for multiple physiological applications requiring temporally aligned multi-site recordings and can enable unobtrusive multi-signal acquisitions without physical interconnections between sensing units, supporting monitoring during free movement and daily-life activities.

Previous works quantified and optimized synchronization and data-flushing performance, identifying a 40 Hz beaconing frequency and a dynamic BLE connection interval as the most effective operating configurations. In controlled laboratory conditions, without direct application on human subjects, the platform achieved a median inter-node synchronization delay of 1.18 ticks (with a 16 MHz system clock) and a min-max range of approximately 100 ns [1], [2]. BLE data transmission was also evaluated, showing robust performance with a proximal gateway, while non-line-of-sight configurations reduced throughput by approximately 30%, highlighting the importance of gateway placement in home-monitoring deployments [3].

This study is designed as a clinically motivated engineering proof of concept to evaluate the performance of the proposed prototype platform in a relevant cardiovascular monitoring scenario, the cuff-less blood pressure monitoring based on pulse arrival time.

From a translational perspective, the aim is to assess whether a fully wireless and synchronized multi-node wearable architecture can reliably capture physiologically meaningful cardiovascular trends under operating conditions compatible with future home and remote monitoring workflows. In this context, the clinical value of the system lies in enabling repeated and unobtrusive multi-signal acquisitions outside conventional laboratory settings, with the potential to support longitudinal cardiovascular assessment, patient stratification, and escalation toward more targeted measurements when abnormal recovery or hemodynamic responses are detected. Accordingly, study outcomes should be interpreted as evidence of physiological monitoring and operational feasibility, while direct clinical adoption for quantitative blood pressure estimation would require dedicated validation against continuous and invasive reference measurement methods.

From a clinical perspective, BP monitoring based on cuff-less approaches aims to overcome limitations of traditional occlusive methods, such as user activity interruption and white-coat effect, by enabling BP assessment during normal daily routines. Indeed conventional oscillometric devices provide only intermittent snapshots, which may be insufficient for clinicians to capture hemodynamic fluctuations occurring outside the clinic. Cuff-less systems, typically based on Pulse Transit Time (PTT) or Pulse Arrival Time (PAT), offer promising alternatives for tracking BP-related cardiovascular dynamics over time while minimizing patient discomfort. PAT is defined as the interval between cardiac depolarization, typically detected via electrocardiography (ECG), and pulse arrival at a peripheral site, typically measured via photoplethysmography (PPG), whereas PTT measures the wave travel time between two arterial locations. Both parameters are inversely correlated with arterial pressure and vascular stiffness [4], [5], [6], as elevated BP accelerates pulse wave propagation. As PAT includes the Pre-Ejection Period (PEP), it is more affected by electromechanical delay than PTT [26]. Nevertheless, in the proposed proof of concept, PAT was selected due to the centrality of ECG and PPG in standard cardiovascular wearable setups, as these two physiological signals represent well-established functional markers in this application domain.

Multiple studies have confirmed significant correlations between PTT or PAT and systolic (SBP) and diastolic (DBP) blood pressure [7], [8], [9], [10], [11], [12], [13], [14], [15], [16], [17], [18], [19], [25].

Several works investigating BP estimation using PAT or PTT have relied on wired systems in controlled laboratory environments to ensure precise signal synchronization, at the expense of wearability, portability, and patient comfort. While these approaches provide valuable physiological insight, their reliance on wired instrumentation limits applicability to continuous and long-term monitoring in everyday settings. In [11], data from 2309 surgical patients collected using wired bedside monitors showed moderate correlations between PAT and SBP ($r \approx -0.37$) and DBP ($r \approx -0.30$). While DBP estimation using PAT met AAMI standards [27], SBP accuracy was lower and influenced by confounding factors. In [12], pharmacological BP modulation via phenylephrine was applied to 30 healthy volunteers to assess the influence of PEP on PAT, using wired ECG and fingertip PPG sensors. The relatively small PEP increase ($\approx 5.5$ ms) compared to the PTT change ($\approx -16.8$ ms) supported the physiological validity of PAT-based approaches. Personalized PAT-based models achieved RMSEs of approximately 5.5/3.8 mmHg for SBP/DBP, only slightly worse than PTT-based estimates. In [18], six PAT definitions derived from ECG and PPG signals collected at different peripheral sites were evaluated in 32 subjects undergoing BP-modulating tasks using a fully wired setup. Toe PAT exhibited the strongest SBP correlation ($r = -0.63 \pm 0.05$), outperforming conventional finger-based measurements.

Other studies have investigated PAT-based BP estimation using integrated wearable devices that embed ECG and PPG sensors into compact body-worn units, typically positioned on the wrist or chest. Although these solutions improve ease of use, they constrain sensor placement and may limit adaptability across different monitoring scenarios or patient needs. In [13], a wearable armband combining arm ECG and PPG achieved accurate SBP estimates ($\mathrm {RMSE} \approx 4.71$ mmHg), meeting AAMI standards when heart rate was incorporated. In [14], a wrist-worn device integrating ECG, seismocardiography (SCG), and multi-wavelength PPG was evaluated over 24 h in 21 subjects, achieving a Pearson correlation of approximately 0.69 with SBP and an RMSE of approximately 2.7 mmHg; with two-point calibration, RMSE increased to approximately 3.9 mmHg. In [15], a chest-worn wireless belt recorded ECG and PPG in 18 cyclists during incremental exercise, revealing strong intra-subject correlations with SBP ($r \approx 0.81$), although DBP correlations were not significant.

Alternative approaches for PTT estimation using signals beyond ECG-PPG have also been explored, though they often require active user participation, limiting unobtrusive use during daily life. In [16], the SeismoWatch device combined SCG acquired via a sternum-pressed accelerometer with wrist PPG in 13 subjects undergoing BP changes. Although DBP estimation accuracy was high ($\mathrm {RMSE} \approx 2.9$ mmHg), manual chest contact reduced usability. In [17], the CareUp smartwatch combined wrist and fingertip PPG signals to estimate PTT via cross-correlation and a linear model including heart rate. Validation on 44 subjects showed performance approaching AAMI standards, but required voluntary finger placement. In [19], an integrated system combining ECG electrodes with a MEMS microphone to capture the mechanical pulse at the radial artery achieved low SBP/DBP estimation errors ($2.72 \pm 3.42 / 2.29 \pm 3.53$ mmHg) in a single-subject study.

Overall, the current state of the art highlights a recurring trade-off across cuff-less BP monitoring systems. Wired systems provide reliable synchronization but are poorly suited for wearable long-term monitoring outside clinical settings. Compact integrated wearables improve usability, but often constrain sensor placement to a single body district and may therefore reduce adaptability across applications or patient conditions. Alternative approaches based on non-standard sensing combinations can achieve promising results, but frequently require active user participation or specific measurement postures. Within this context, the proposed platform addresses a main engineering challenge: enabling synchronized acquisition from distributed sensing nodes positioned at anatomically distinct sites, while preserving a fully wireless and modular architecture suitable for multiple remote monitoring scenarios.

This work is framed as an engineering proof of concept to evaluate the proposed multi-purpose wireless platform in a realistic and clinically motivated scenario. Two autonomous platform sensing nodes were paired with two low-cost commercial front-ends to synchronously collect ECG and PPG signals for PAT estimation during modest hemodynamic changes, with the aim of quantitatively assessing BP variations in response to physiological perturbations. The platform setup integrates three main components: (1) a chest-worn ECG node, (2) a wrist-worn PPG node, and (3) a Raspberry Pi-based gateway for data aggregation. Sub-microsecond synchronization, ensured by the proprietary protocol, provides a stable timing reference for accurate signal acquisition at different body locations. Acquired signals are then streamed to the gateway and subsequently processed offline for artifact removal, beat detection, and PAT extraction.

The experimental campaign involved 19 healthy adults (12 men and 7 women, aged 23-57 years) without known cardiovascular conditions. The protocol simulated a moderate exercise session representative of a home-monitoring context, inducing transient BP changes. ECG and PPG signals were recorded before and after exercise, while reference BP was measured during the same period using a validated digital sphygmomanometer.

The comparison between PAT, computed from signals collected with the proposed platform, and reference BP values was designed to assess whether the fully wireless, time-synchronized multi-node architecture can reliably capture physiologically meaningful cardiovascular trends in a controlled proof-of-concept scenario, while maintaining unobtrusive operating conditions compatible with remote monitoring. The present study does not aim to establish clinical generalizability or absolute replacement of standard blood pressure measurements, but rather to verify the feasibility of the proposed engineering framework as a basis for future translational developments. Overall, the results support the potential of the proposed platform for longitudinal cardiovascular monitoring and its applicability in remote and home-based clinical contexts.

The structure of this paper is organized as follows. Section II details the platform architecture, the PAT estimation strategy, and the experimental campaign; Section III presents the results and statistical analyses; Section IV discusses the findings, limitations, and future directions.

## II. Methods and Procedures

### A. Prototype Platform Overview

**FIGURE 1.**
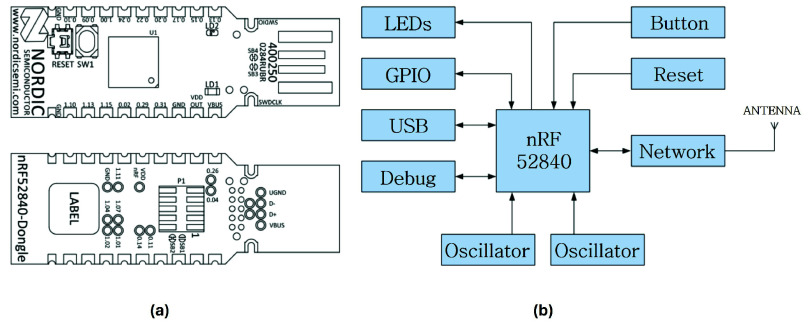
(a) Front and rear views of the nRF52840 USB Dongle. (b) Schematic representation of the peripheral components integrated with the nRF52840 SoC.

The wearable system is composed of multiple autonomous sensing nodes within a fully wireless architecture, designed to minimize encumbrance and allow unobtrusive use during daily activities. Each node is based on Nordic Semiconductor’s nRF52840 USB Dongle, which integrates a 32-bit ARM Cortex-M4 processor, 1 MB flash memory, 256 KB RAM, a 12-bit ADC, and a 2.4 GHz transceiver supporting Bluetooth Low Energy (BLE) 5.4, BLE Mesh, Thread, Zigbee, ANT, and proprietary protocols (Fig. 1). To form a sensing unit, the selected System-on-Chip (SoC) is paired with an application-specific analog transducer, enhancing system modularity and adaptability to different physiological signals. Once configured, each node is able to sample and locally store biosignals for a defined acquisition window, depending on available memory resources.

To support continuous monitoring beyond the limited on-board storage capacity of the SoC, a Raspberry Pi 3 was employed as a data collector and gateway (quad-core Broadcom BCM2837 at 1.2 GHz, 1 GB RAM, integrated Wi-Fi and Bluetooth interfaces). Sensing nodes periodically transmit buffered data via BLE to the gateway, which runs a lightweight TCP server to forward biosignals to an external workstation for analysis. Alternatively, signal processing and feature extraction algorithms may be deployed directly on the gateway to enable a fully on-edge solution in future developments.

**FIGURE 2.**
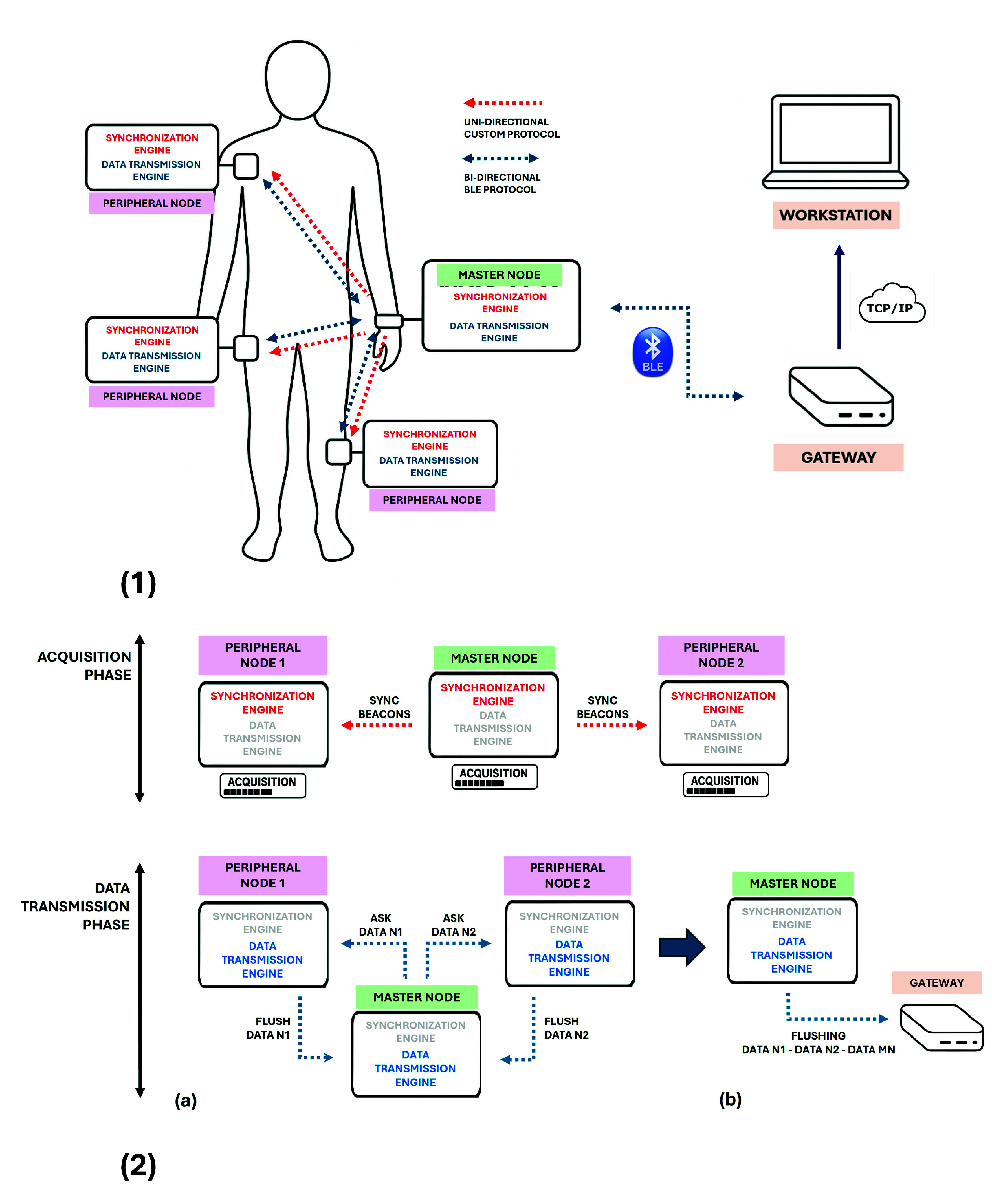
(1) System-level overview. Each sensing unit is built around the nRF52840 SoC paired with application-specific analog front-ends. A Raspberry Pi 3 aggregates biosignals and forwards data to an external workstation. Two software layers support operation: a custom synchronization engine for time-aligned sampling and a BLE-based data transmission engine. (2) Operation cycle of the dual-protocol architecture. During the acquisition phase, the MN broadcasts synchronization packets to enable time-aligned sampling. During the transmission phase, SNs stream buffered data to the MN (a), which then forwards the aggregated dataset to the gateway via BLE (b).

Architecturally, the platform relies on a dual-protocol strategy that decouples precise temporal alignment from data transmission (Fig. 2). All nodes participate in a standard BLE network, where one sensing unit is designated as Master Node (MN) and operates as BLE central, collecting data from the remaining Slave Nodes (SNs) acting as BLE peripherals and streaming the entire data flow to the gateway. In parallel, the MN broadcasts synchronization beacons over a lightweight proprietary 2.4 GHz protocol, sharing the same radio hardware used by the BLE stack and forming an intra-platform synchronization mesh network. This broadcast-based synchronization network aligns the local clocks of all SNs to the MN time base with sub-microsecond accuracy, while minimizing latency and jitter through reduced protocol overhead.

The proposed platform is robust by design with respect to increases in the number of deployed sensing nodes, which scale with the number and type of biosignals required by the target application. Indeed, the BLE network is designed to support multiple nodes, while the broadcast-based synchronization mechanism is inherently independent of node count, enabling extensibility to multi-modal configurations.

A typical platform operation cycle alternates between acquisition and data transmission phases (Fig. 2). During the acquisition phase, all sensing nodes simultaneously sample their respective biosignals based on a shared time reference provided by MN through the synchronization engine. Indeed synchronization packets are periodically broadcast by the MN, ensuring temporal alignment of ADC triggering events across distributed nodes. Once the acquisition window terminates, after a configurable duration, the platform enters the transmission phase, which is structured as a two-step process. First, each SN transmits its locally buffered data to the MN that, upon collecting the full dataset from all peripheral nodes, subsequently forwards the aggregated dataset to the external gateway. This separation of concerns, custom protocol for synchronization and BLE for data transfer, enables deterministic sampling with sub-microsecond alignment and also leverages BLE efficiency for flexible and energy-efficient data streaming.

Previous studies on the proposed platform [1], [2], [3] demonstrated that this dual-protocol architecture can achieve sub-microsecond synchronization accuracy together with reliable wireless data throughput. In controlled laboratory conditions, without interaction with human subjects, the configuration using a 40 Hz synchronization beacon frequency and dynamic BLE connection interval adaptation achieved a median inter-node synchronization delay of 1.18 clock ticks (with a 16 MHz system clock), with a min-max range of approximately 100 ns [1], [2]. Data transmission performance was also evaluated under increasing environmental complexity, showing robust operation with a proximal gateway and a throughput reduction of approximately 30% in non-line-of-sight configurations [3]. Overall system throughput was quantified through the Efficiency Index (a dimensionless metric ranging between 0 and 1, with 0 indicating an infinite data flushing time and 1 indicating instant data transfer), which reached approximately 0.35 in proximity-gateway configurations [3]. This implies that 35% of the defined acquisition cycle can be used for acquisition, while the remaining 65% represents a “blind” period required for data transmission.

### B. Synchronized Acquisitions and Pulse Arrival Time Extraction

This study is the first application of the prototype system in a real-world monitoring scenario. The research was designed as a proof of concept to assess the feasibility of a PAT-based cuff-less blood pressure monitoring approach using physiological signals synchronously acquired with the proposed platform. Two sensing units were employed: an ECG node positioned on the chest to record cardiac electrical activity and a wrist-worn PPG node to detect peripheral pulse waves. Their synchronized acquisition enables beat-by-beat PAT extraction and analysis of cardiovascular responses to physical perturbations.

PAT is defined as the temporal interval between the R-peak of the ECG and a fiducial point on the subsequent peripheral PPG waveform. It includes both a cardiac component, the pre-ejection period (PEP), and a vascular component, the pulse transit time (PTT). PTT is a more specific vascular marker, while PAT is very convenient to adopt in wearable applications due to the accessibility and robustness of ECG and PPG signals. Although PAT deviations are on the order of milliseconds, continuous inter-node alignment is considered an enabling condition for this application, as it ensures that clock drift phenomena remain negligible over time with respect to physiological variability and do not accumulate over prolonged monitoring sessions.

**FIGURE 3.**
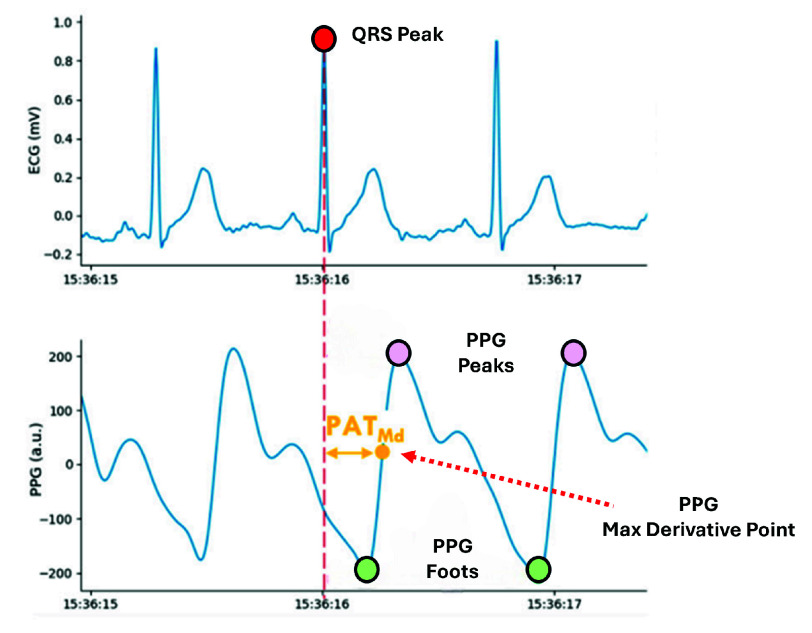
Pulse Arrival Time definition. $PAT_{Md}$ is computed as the delay between the ECG R-peak (red) and the maximum slope point of the PPG waveform (yellow).

In this study, for each denoised cardiac cycle a PAT value is computed by measuring the delay between the extracted R-peak in the ECG and the computed point with the maximum first derivative in the following PPG waveform ($PAT_{Md}$, Fig. 3). $PAT_{Md}$ has been shown in the literature to correlate most robustly with blood pressure and is often used as the primary timing indicator, since this PPG fiducial point ($PPG_{Md}$) is less affected by wave reflection artifacts and peripheral vascular characteristics [12], [20].

**FIGURE 4.**
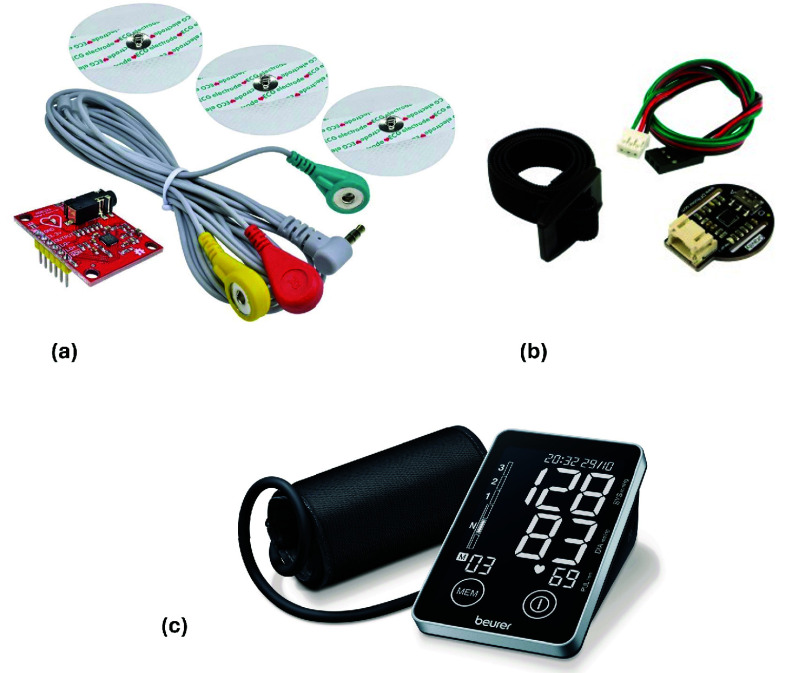
Sensing components. (a) ECG analog front-end with electrodes. (b) Wrist-worn reflective PPG band. (c) Reference oscillometric BP device (Beurer BM 58).

We adopted two different analog transducers and paired them with two platform nodes to build the sensing units required for the specific case study (Fig. 4). The single-lead ECG signal was collected using a commercial analog front end based on the AD8232 chipset (Analog Devices), with three electrodes placed according to a standard single-lead configuration (RA, LA, LL). The PPG signal was acquired using a commercial reflective wristband based on the SON1303 chipset (Soon Electronic), worn over the radial artery of the right wrist. The module integrates a green LED emitter and a photodiode receiver to implement reflective photoplethysmography and capture the analog pulse waveform. Both analog signals were digitized via the on-board analog-to-digital converters (ADCs) of the platform nodes, while a Beurer BM 58 oscillometric device was used to measure reference BP values.

**FIGURE 5.**
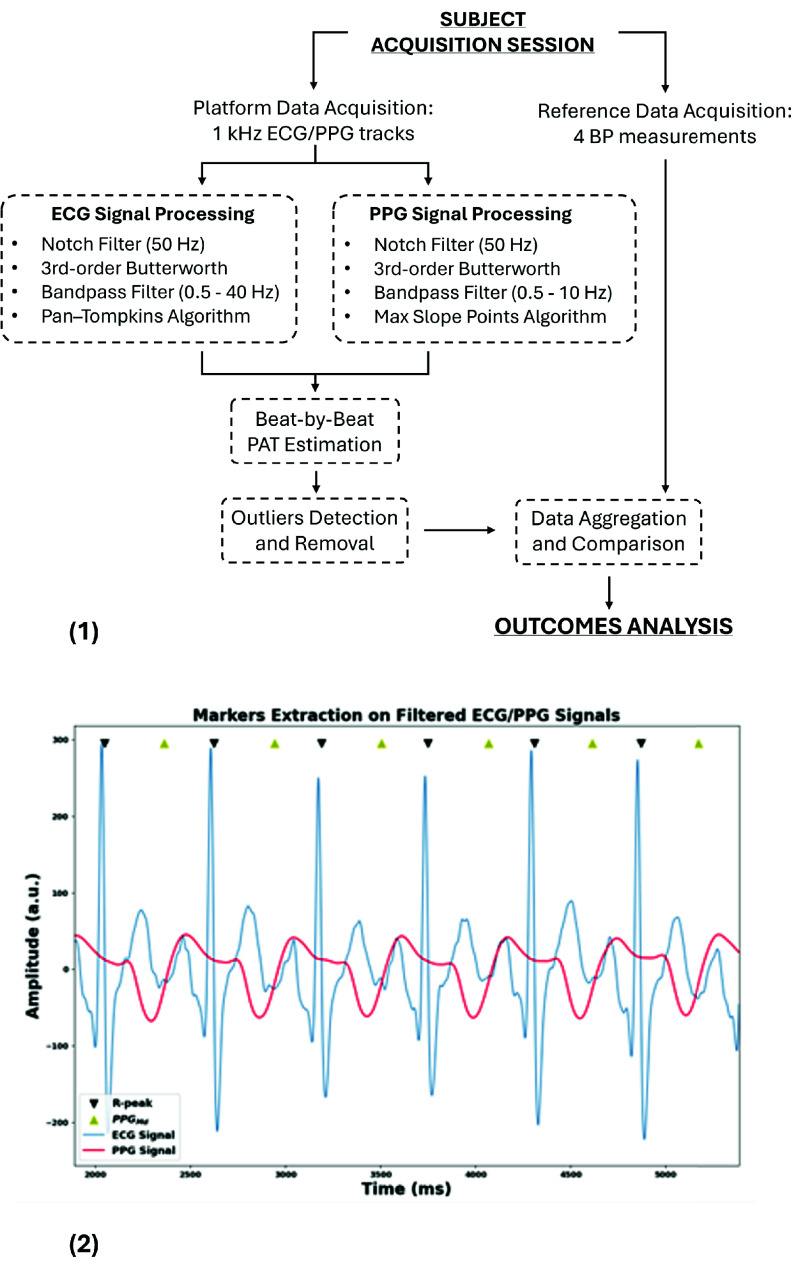
(1) Signal processing workflow. ECG, PPG, and BP measurements are processed in parallel and then compared for statistical analysis. (2) Signal processing example: filtered ECG and PPG signals with detected fiducial points, $R_{peak}$ (black) and $PPG_{Md}$ (yellow) markers.

Analog signals were sampled at 1 kHz by the platform, stored in the gateway and then processed offline on the workstation (Python 3.8.20, Fig. 5). A notch filter was applied to suppress power-line noise (50 Hz). ECG was then bandpass filtered (0.5 - 40 Hz) to isolate QRS complexes, whereas the PPG filter was implemented with 0.5 and 10 Hz as band limits to emphasize pulsatile components and suppress motion artifacts. Specifically, both signals were processed with a 3rd-order Butterworth bandpass filter. ECG R-peaks were then detected with a Pan-Tompkins implementation, while $PPG_{Md}$ markers were identified as the max slope points between each $R_{peak}$ and the subsequent PPG peak (located via a local maxima detection algorithm). This workflow enabled beat-by-beat analysis by isolating fiducial markers in each cardiac cycle (Fig. 5). Finally, $PAT_{Md}$ was estimated as the time delay between the two markers for each heartbeat.

For each subject, two acquisition sessions (composed of 11 consecutive acquisition cycles) were performed, one before and one after the blood pressure perturbation exercise, with each cycle recording 10 s of data. The median $PAT_{Md}$ value across detected beats was retained for each cycle. For each session, a rolling-window filter with 3-elements width detected and excluded outliers. For each value in the time series of median $PAT_{Md}$ values, the mean and standard deviation of the window centered on it were computed: if the point deviated from the local mean by more than three times the standard deviation, it was considered an outlier and discarded; otherwise, it was retained. Concurrently, reference BP values (SBP and DBP) were manually recorded during each session after acquisition cycles 1, 4, 7, and 10, ensuring temporal alignment with the PAT time series for subsequent analyses.

### C. Experimental Campaign

We designed and carried out an experimental campaign with a population of subjects undergoing a blood pressure perturbation in order to evaluate the performance of the system in effectively tracking the induced hemodynamic changes through PAT estimation.

A total of 19 healthy adults (aged 23-57 years, both sexes) without known cardiovascular conditions were recruited for the experimental protocol. All subjects completed a brief questionnaire on anthropometrics and lifestyle (Table 1). All experimental procedures were conducted in accordance with the principles of the Declaration of Helsinki and received formal approval from the Ethics Committee of Politecnico di Milano (Opinion No. 44, December 13, 2023), and all participants provided written informed consent.

**TABLE 1:** Subjects anthropometric data and lifestyle characteristics.

	All (19 Subjects)		Men (12 Subjects)		Women (7 Subjects)	
Metric	Mean	Std	Mean	Std	Mean	Std
Age (years)	31	9	34	10	27	4
Height (cm)	173.0	9.1	177.3	8.6	165.6	3.4
Weight (kg)	69.7	12.2	74.7	11.3	61.1	8.7
BMI	23.2	3.3	23.8	3.5	22.3	2.7
Smoking (Y/N)	3/19		2/12		1/7	
Physical activity (Y/N)	9/19		7/12		2/7	

**FIGURE 6.**
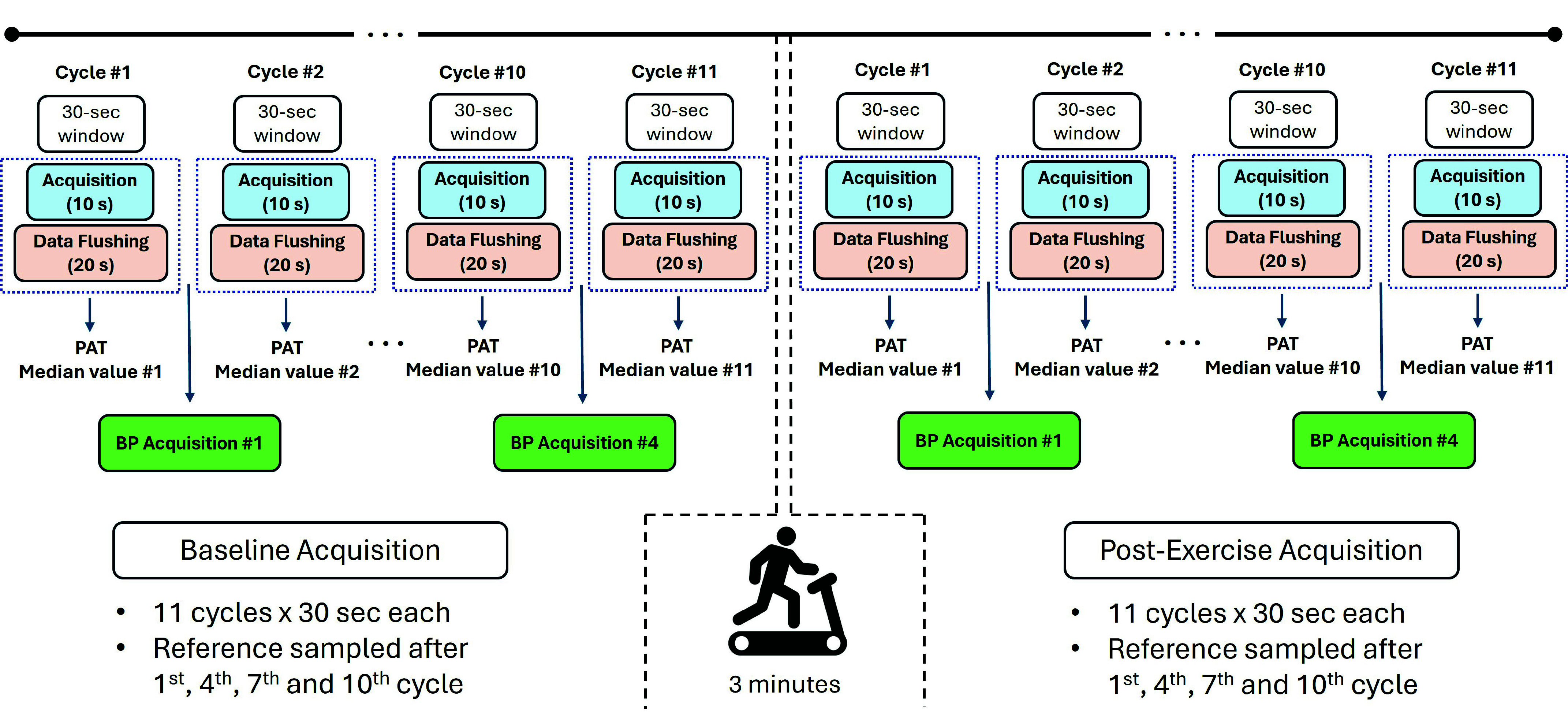
Experimental protocol timeline: 6-minute baseline acquisition, 3-minute treadmill exercise, and 6-minute post-exercise acquisition. Each session includes 11 consecutive acquisition cycles and four reference BP measurements.

The protocol was designed to assess platform reliability in detecting BP variations during a controlled cardiovascular perturbation induced by moderate physical activity (Fig. 6). The exercise stimulus was deliberately set to low-moderate intensity to emulate standard daily-life physical exertion, consistent with the proposed remote home-monitoring use case, and to minimize any cardiovascular risk since many subjects were not used to regular exercise. Before data collection, participants completed a brief questionnaire about perceived physical tolerance; based on their responses, 3-min running at 7.5 km/h was selected as sustainable across the population.

Each subject was first instrumented with the full acquisition setup. This included ECG electrodes and the chest sensing unit composed of an AD8232 module linked to a platform node, a wrist-worn PPG band equipped with a sensing unit composed of a SON1303 module linked to another platform node, and the cuff-based BP monitor on the opposite arm. Functionally, the ECG node served as the platform Master Node, while a nearby Raspberry Pi handled BLE data reception as the platform gateway.

The baseline acquisition session included 11 consecutive acquisition cycles. Considering the platform’s Efficiency Index [3], each cycle consisted of 10 s of synchronized ECG and PPG recordings and a subsequent 20 s data transfer phase. Sphygmomanometer readings were taken after cycles 1, 4, 7, and 10. With each cycle lasting approximately 30 s, this ensured more than 90 s between consecutive cuff BP measurements, meeting clinical guidelines for avoiding cuff-induced artifacts [21], [22]. Reference BP values were sampled intermittently rather than continuously, in order to limit repeated cuff inflations and preserve a protocol compatible with a realistic wearable monitoring scenario. Consequently, the validation outcomes focused on trend comparison across physiological phases and recovery trajectories rather than on beat-to-beat equivalence. The total duration of the baseline acquisition session was 6 minutes.

Subsequently, participants performed 3-minute treadmill exercise at 7.5 km/h to transiently increase BP without excessive strain. Upon completion, the subject was re-instrumented using the same sensor placement, and a second acquisition session was conducted under identical conditions to capture both the immediate post-exercise state and the recovery dynamics of the cardiovascular system. This second 6-minute session also included 11 consecutive acquisition cycles and four reference BP measurements at the same time points. After completion of the post-exercise acquisition session, the subject was released from all instrumentation.

## III. Results

Data collected for each subject were aggregated on the external workstation and processed offline according to the workflow described in Fig. 5.

**FIGURE 7.**
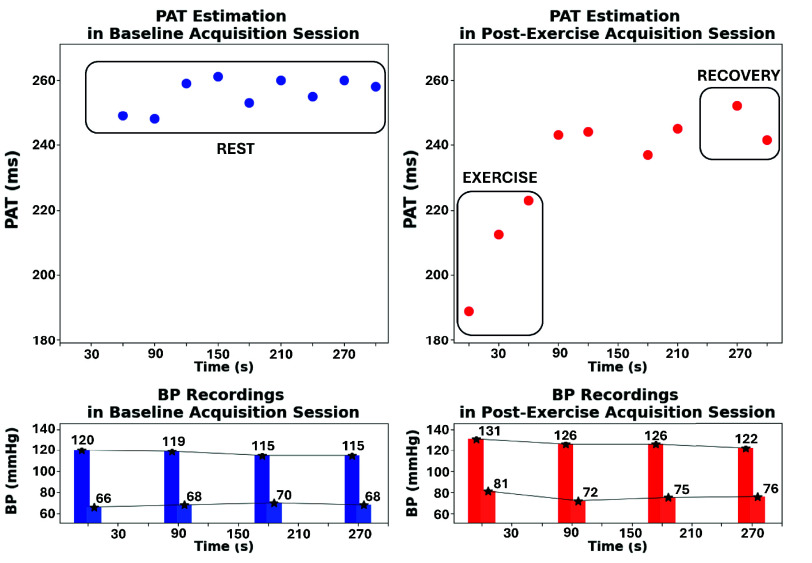
PAT and BP data from a representative subject. Top: median PAT values collected during baseline (left) and post-exercise (right) sessions. Bottom: corresponding SBP and DBP values from the reference device.

A total of 418 acquisition windows were analyzed, corresponding to 19 subjects completing two sessions (baseline and post-exercise), each composed of 11 acquisition cycles. Each 30-second cycle (10 s acquisition and 20 s transmission) provided a single median PAT value used in subsequent analyses. To mitigate the impact of motion artifacts and occasional low-quality segments, an outlier detection and removal step was applied to each subject’s PAT time series using a local rolling-window strategy, as described in Section II. An example of the elaboration outcome for a representative subject is shown in Fig. 7. The upper panels display the median PAT values (in milliseconds) extracted from each 10-second acquisition window during the baseline acquisition session (left) and the post-exercise acquisition session (right). The recordings are grouped into three physiological phases. All acquisitions from the baseline session correspond to the REST phase, where subjects have not yet experienced the physiological perturbation. During the post-exercise session, acquisitions #1-3 were performed immediately after the physical effort and represent the EXERCISE phase, representative of peak cardiovascular stress. Acquisitions #9-11 correspond to the RECOVERY phase, after a progressive normalization. Each point corresponds to a valid PAT estimation, while missing entries indicate data excluded by the outlier rejection algorithm. The lower panels show the reference SBP and DBP values obtained from the oscillometric device, recorded after cycles #1, 4, 7, and 10 in both sessions.

**FIGURE 8.**
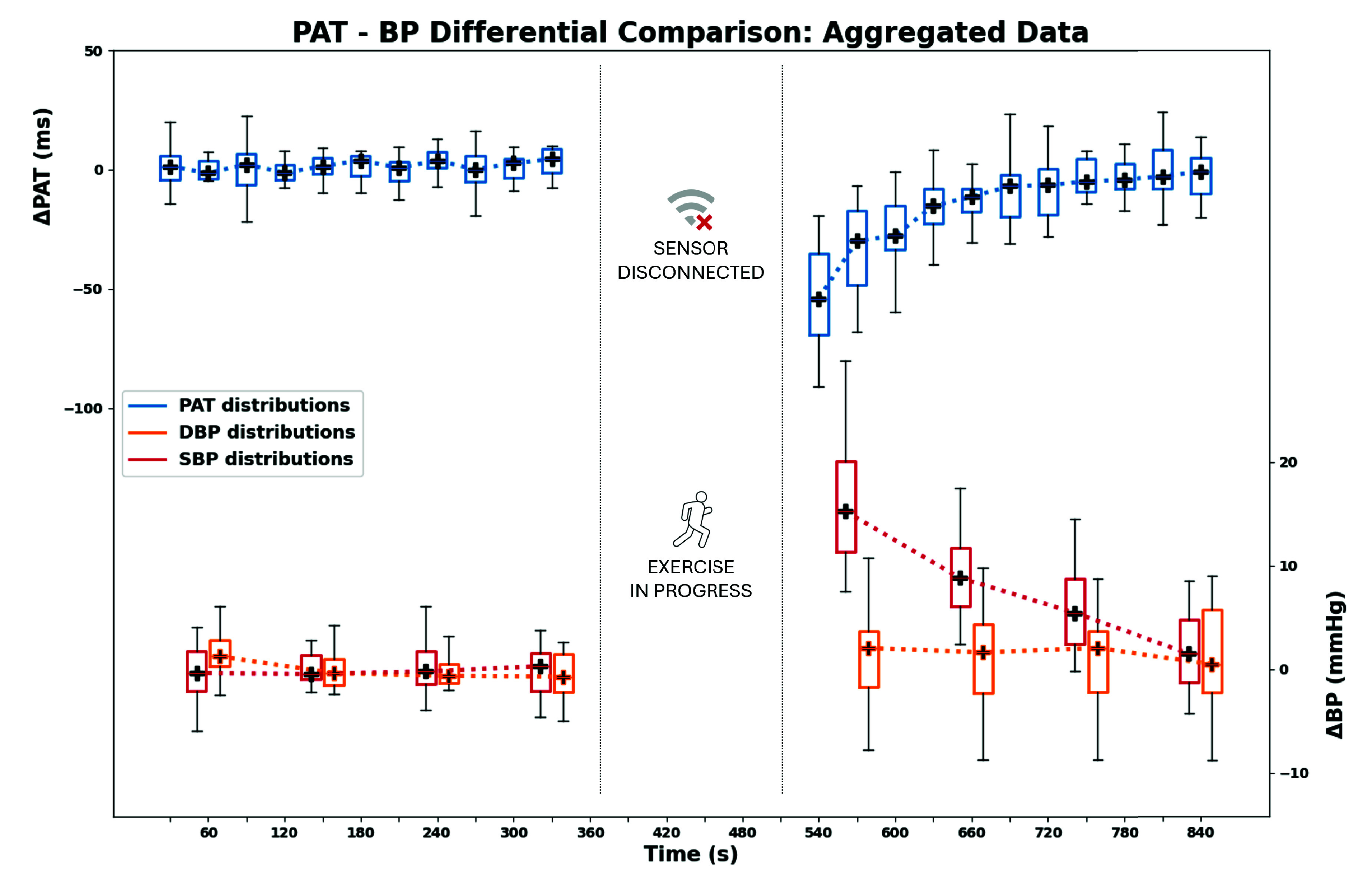
Temporal evolution of relative PAT, SBP and DBP distributions. Physical exercise separates baseline and post-exercise acquisition sessions.

Acquired data from all participants were then aggregated to generalize the analysis across the population. While absolute values retain physiological meaning, they reflect individual anatomy and sensor placement variability. To enable cross-subject aggregation, relative PAT series were computed for each subject by subtracting the average baseline value from both baseline and post-exercise time series. This normalization removes inter-subject offsets while preserving intra-subject dynamics. A similar approach was applied to SBP and DBP data from the reference device: BP series of each subject were centered around their baseline to obtain relative BP variations. Fig. 8 presents the normalized aggregated dataset over time. Boxplots show the distributions across the population of relative PAT (top) and BP values (bottom). In both sessions, PAT values are grouped per acquisition cycle, while BP values were measured after cycles #1, 4, 7, and 10.

**FIGURE 9.**
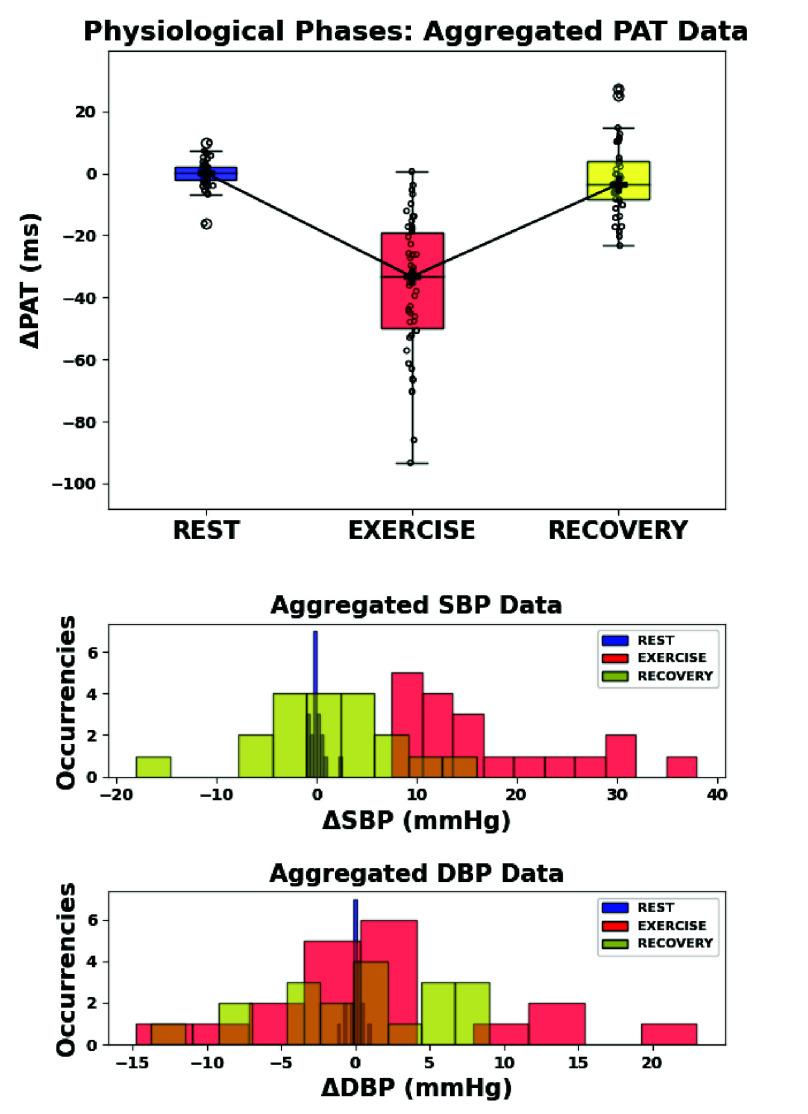
Aggregated phase-wise distributions. Top: PAT values show a V-shape pattern over time. Bottom: SBP increases during EXERCISE and then recovers; DBP remains relatively stable.

Fig. 9 further aggregates relative PAT and BP distributions across the three physiological phases (REST, EXERCISE, RECOVERY). For each subject, REST BP values were computed by averaging the four recordings during the baseline session, while EXERCISE and RECOVERY BP values were respectively the first and the last recording during the post-exercise session. As expected, PAT drops during EXERCISE and gradually rises in RECOVERY, showing a characteristic V-shape pattern. The lower panels present the aggregated blood pressure measurements. SBP exhibits a modest but statistically significant increase after exercise, followed by a progressive return toward baseline during recovery, consistent with the protocol’s aim to model small and transient hemodynamic perturbations representative of daily-life exertion. DBP remains comparatively stable.

To statistically assess whether the distributions in Fig. 9 differed across physiological phases, each distribution (PAT, SBP, DBP) was first tested for normality via the Shapiro-Wilk test (Table 2). PAT and SBP violated normality in multiple phases, while DBP passed all tests. Consequently, a non-parametric strategy was adopted. The Kruskal-Wallis test revealed highly significant differences across phases for PAT ($H = 86.835$, $p = 1.39 \times 10^{-19}$) and SBP ($H = 34.612$, $p = 3.05 \times 10^{-8}$), while no significant effect was observed for DBP ($H = 0.651$, $p = 0.722$) (Table 3). Mann-Whitney post-hoc tests (with Bonferroni correction) showed significant differences comparing REST-EXERCISE and EXERCISE-RECOVERY phases for both PAT and SBP ($Adj.\ p < 0.0001$), confirming a marked hemodynamic response to the exercise perturbation (Tables 4 and 5). No significant difference was observed between REST and RECOVERY, indicating a trend back toward baseline. DBP was excluded from post-hoc testing due to the absence of significant Kruskal-Wallis results.

**TABLE 2:** Shapiro–Wilk test across physiological phases.

Phase	PAT $p$	SBP $p$	DBP $p$
REST	0.0140	0.0054	0.0926
EXERCISE	0.0001	0.0286	0.3159
RECOVERY	0.4100	0.7073	0.4211

**TABLE 3:** Kruskal–Wallis test across physiological phases.

Variable	H Statistic	p-value
PAT	86.835	$1.39 \times 10^{-19}$
SBP	34.612	$3.05 \times 10^{-8}$
DBP	0.651	0.722

**TABLE 4:** Post-hoc Mann–Whitney test for PAT.

Comparison	U	Raw p	Adj. p	Sig.
REST vs EXERCISE	2799.5	< 0.0001	< 0.0001	Yes
EXERCISE vs RECOVERY	123.0	< 0.0001	< 0.0001	Yes
REST vs RECOVERY	1386.5	0.0714	0.2142	No

**TABLE 5:** Post-hoc Mann–Whitney test for SBP.

Comparison	U	Raw p	Adj. p	Sig.
REST vs EXERCISE	0.001	< 0.0001	< 0.0001	Yes
EXERCISE vs RECOVERY	342.0	< 0.0001	< 0.0001	Yes
REST vs RECOVERY	137.0	0.2089	0.6267	No

To model the temporal evolution of PAT during recovery at the population level, a curve fitting analysis was conducted on aggregated post-exercise data (Fig. 8). The aim was to compare the suitability of three parametric models in describing the aggregated PAT recovery trend: a mono-exponential function (Eq. 1), widely used to characterize first-order autonomic recovery [23], [24], a linear function (Eq. 2) and a logarithmic function (Eq. 3).

\begin{align*} \mathrm {PAT}(x)& = \mathrm {PAT}_{\infty }+ \Delta \mathrm {PAT} \cdot e^{-k(x - 1)} \tag {1}\\ \mathrm {PAT}(x)& = a \cdot x + b \tag {2}\\ \mathrm {PAT}(x) & = a + b \cdot \log (x) \tag {3}\end{align*}

**FIGURE 10.**
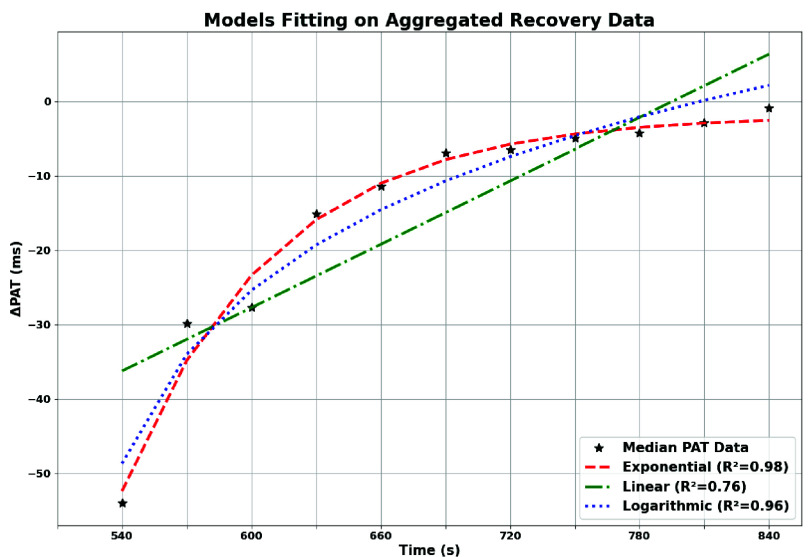
Fitting comparison on aggregated PAT recovery trend. Mono-exponential and logarithmic models achieve a stronger agreement with experimental data compared to the linear model.

Fitting was performed on the median values of the aggregated PAT distributions within the post-exercise session, and model performance was assessed using the coefficient of determination $R^{2}$. As shown in Fig. 10, the mono-exponential model best captured the observed recovery dynamics, modeling the initial drop followed by progressive normalization. The logarithmic model provided a comparable but slightly less accurate fit, particularly in the early stages. The linear fit approximated the overall trend but showed a lower correspondence. Estimated model parameters and associated $R^{2}$ values are summarized in Table 6. The mono-exponential model achieved the highest agreement ($R^{2} = 0.98$), supporting its suitability to describe the recovery trend observed in this proof-of-concept scenario.

**TABLE 6:** Fitting results on aggregated PAT recovery data: model parameters and coefficient of determination $R^{2}$.

Model	Parameter	Symbol	Value	$R^{2}$
Mono-exponential	Asymptote	$\mathrm {PAT}_{\infty } $	-1.77 ms	0.980
	Initial excursion	$\Delta \mathrm {PAT}$	-50.62 ms	
	Decay constant	$k$	0.43	
Linear	Slope	$a$	4.27	0.764
	Intercept	$b$	-40.50	
Logarithmic	Offset	$a$	-48.69	0.955
	Scaling factor	$b$	21.23

The main outcome of the comparison with the gold standard for BP monitoring was the correlation analysis between PAT values extracted from the proposed platform and SBP values recorded by the reference device. Two Recovery Index metrics were computed ($RI_{\mathrm {PAT}}$ and $RI_{\mathrm {SBP}}$) to compare recovery trends described by PAT and SBP. Both indices were normalized per subject using a range-based transformation, where the maximal perturbation (post-exercise minimum for PAT, maximum for SBP) was set to −100%, and the baseline average value was mapped to 0%. This transformation enables direct trend comparison in a shared normalized space, in order to assess if the same physiological condition may be described by the two metrics in a statistically similar way. The corresponding formulations are:

\begin{align*} RI_{\mathrm {PAT}}(i)& = \frac {\mathrm {PAT}(i) - \mathrm {PAT}_{\min }}{\mathrm {PAT}_{\mathrm {baseline}} - \mathrm {PAT}_{\min }} \cdot 100 \tag {4}\\ RI_{\mathrm {SBP}}(i) & = \frac {\mathrm {SBP}(i) - \mathrm {SBP}_{\max }}{\mathrm {SBP}_{\mathrm {baseline}} - \mathrm {SBP}_{\max }} \cdot 100 \tag {5}\end{align*}

where $\mathrm {PAT}(i)$ and $\mathrm {SBP}(i)$ are the values at the $i$-th post-exercise acquisition cycle, $\mathrm {PAT}_{\min }$ and $\mathrm {SBP}_{\max }$ correspond to the minimum and maximum values in the post-exercise session (maximum perturbation), and $\mathrm {PAT}_{\mathrm {baseline}}$ and $\mathrm {SBP}_{\mathrm {baseline}}$ correspond to the baseline-session average values for the same subject.

**FIGURE 11.**
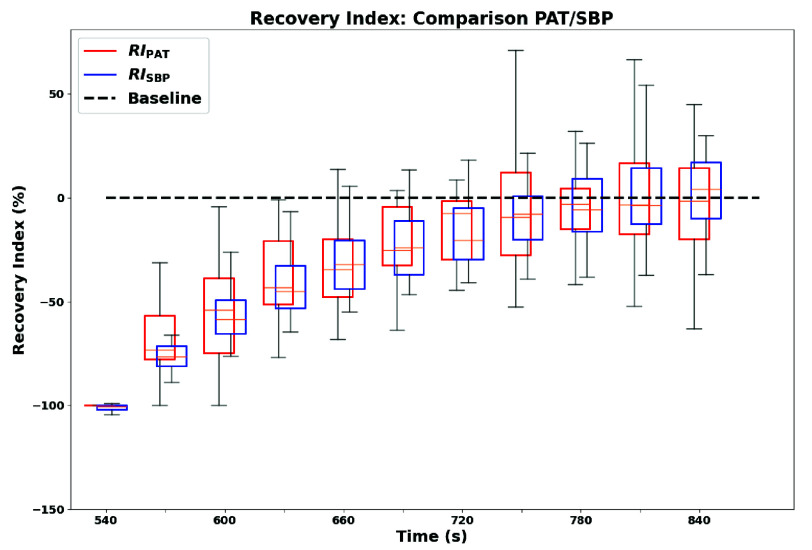
Temporal evolution of $RI_{\mathrm {PAT}}$ and $RI_{\mathrm {SBP}}$ during post-exercise recovery. Baseline corresponds to 0% (black dotted line).

Each subject’s post-exercise session included by design up to $11~{RI}_{\mathrm {PAT}}$ values (if no outliers were detected) but only four ${RI}_{\mathrm {SBP}}$ values. To enable a point-wise comparison of the two metrics over a common time base, $11~{RI}_{\mathrm {SBP}}$ values were derived from the 4-point time series by oversampling using the mono-exponential model previously validated and described in Eq. 1. This procedure aligned the time series of the two indices cycle-by-cycle for each subject. Fig. 11 presents the aggregated distributions of $RI_{\mathrm {PAT}}$ (red) and $RI_{\mathrm {SBP}}$ (blue) across the population, showing similar post-exercise recovery profiles.

For each subject, Pearson’s correlation coefficient ($r$) and associated $p$-value were calculated between the two indices. Table 7 reports the subject-wise Pearson coefficients and corresponding $p$-values computed over the aligned recovery indices.

**TABLE 7:** Subject-wise Pearson correlation coefficients and $p$-values between PAT and SBP recovery indices.

Subject	$r$	$p$	Subject	$r$	$p$
#1	0.92	$6.9{\times }10^{-5}$	#11	0.98	$9.3{\times }10^{-8}$
#2	0.95	$5.2{\times }10^{-6}$	#12	0.75	$7.9{\times }10^{-3}$
#3	0.97	$4.1{\times }10^{-7}$	#13	0.95	$8.6{\times }10^{-6}$
#4	0.96	$3.6{\times }10^{-6}$	#14	0.50	$1.2{\times }10^{-1}$
#5	0.61	$4.6{\times }10^{-2}$	#15	0.91	$9.8{\times }10^{-5}$
#6	0.93	$3.5{\times }10^{-5}$	#16	0.97	$6.0{\times }10^{-7}$
#7	0.85	$8.5{\times }10^{-4}$	#17	0.94	$1.9{\times }10^{-5}$
#8	0.90	$1.8{\times }10^{-4}$	#18	0.97	$1.3{\times }10^{-6}$
#9	0.96	$4.4{\times }10^{-6}$	#19	0.97	$6.0{\times }10^{-7}$
#10	0.96	$1.6{\times }10^{-6}$			

Subjects #5 and #14 exhibited the lowest correlation values in the population ($r =0.61$ and $r =0.50$, respectively), with Subject #14 also showing a non-significant p-value. To summarize the population-level correlation more robustly, we inspected the distribution of subject-wise Pearson coefficients and identified these two values as moderate outliers using a z-score criterion ($|z| > 2$). They were therefore excluded from the computation of the population-level mean and standard deviation of the Pearson coefficients to avoid influence of atypical correlation profiles on the descriptive summary of a limited population.

The correlation analysis showed an average Pearson’s coefficient $r$ of 0.93 with a standard deviation of 0.06. The corresponding $p$-value distribution presented an average value of $5.4 \times 10^{-4}$ and a standard deviation of $1.8 \times 10^{-3}$. Notably, all remaining subjects reported $p$-values below the conventional significance threshold of $p < 0.05$, supporting the robustness of the correlation.

**FIGURE 12.**
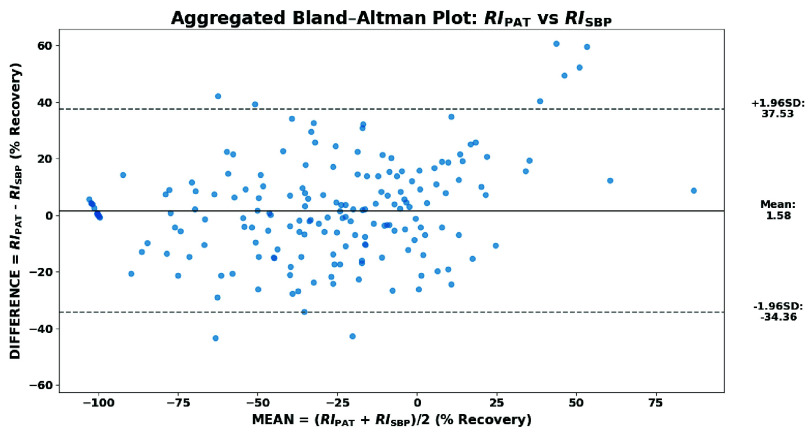
Bland-Altman plot comparing $RI_{\mathrm {PAT}}$ and $RI_{\mathrm {SBP}}$.

To complement the correlation analysis and assess point-wise agreement between PAT and SBP recovery indices, a Bland-Altman analysis was conducted. Unlike Pearson’s coefficient, which captures linear association, the Bland-Altman method quantifies systematic bias and the degree of point-wise interchangeability. For each paired $RI_{\mathrm {PAT}}$ and $RI_{\mathrm {SBP}}$ values, the mean $M_{i} = \frac {RI_{\mathrm {PAT}} + RI_{\mathrm {SBP}}}{2}$ and the difference $D_{i} = RI_{\mathrm {PAT}} - RI_{\mathrm {SBP}}$ were computed. The average bias $\bar {D}$ and limits of agreement (LoA) $\bar {D} \pm 1.96 \cdot \sigma _{D}$ define the expected 95% variation range. The analysis included all valid post-exercise pairs, excluding outlier subjects #5 and #14. The aggregated plot (Fig. 12) showed a small bias (1.58%) but wide LoA (−34.36% / 37.53%), indicating general alignment of recovery trends but notable inter-subject variability. Table 8 reports the individual biases and LoA.

**TABLE 8:** Subject-wise Bland-Altman analysis: $RI_{\mathrm {PAT}}$ vs $RI_{\mathrm {SBP}}$.

Subject	Bias	SD	LoA [low, high]
#1	3.12	12.71	[–21.78, 28.02]
#2	6.75	9.92	[–12.70, 26.20]
#3	34.16	18.91	[–2.90, 71.23]
#4	–0.92	14.27	[–28.89, 27.05]
#5	–	–	–
#6	–15.94	11.05	[–37.61, 5.73]
#7	–5.70	11.77	[–28.78, 17.37]
#8	–1.80	14.39	[–30.01, 26.41]
#9	21.66	10.13	[1.81, 41.52]
#10	11.59	15.03	[–17.87, 41.04]
#11	–1.56	5.80	[–12.92, 9.81]
#12	–22.85	23.91	[–69.71, 24.01]
#13	–7.13	12.21	[–31.06, 16.79]
#14	–	–	–
#15	5.07	11.80	[–18.07, 28.21]
#16	–6.98	8.55	[–23.73, 9.77]
#17	6.01	9.62	[–12.84, 24.86]
#18	7.80	9.00	[–9.84, 25.44]
#19	–6.34	10.00	[–25.93, 13.26]

To unify agreement and correlation into a single metric, the Concordance Correlation Coefficient (CCC) was also computed. The result ($\rho _{c} = 0.885$) confirms strong overall concordance between the PAT and SBP recovery indices. This outcome indicates that PAT and SBP recovery trajectories exhibit a similar temporal evolution, although individual differences can limit point-wise interchangeability.

**FIGURE 13.**
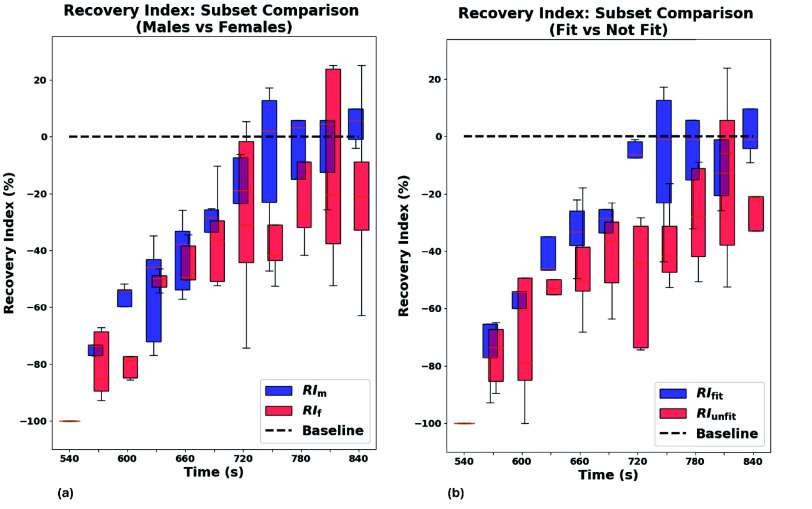
Subgroup comparison of $RI_{\mathrm {PAT}}$ trajectories. (a) Males vs Females (b) Active subjects vs Sedentary ones.

Finally, to begin exploring potential sources of inter-subject variability that may be relevant when considering deployment in heterogeneous real-world populations, a preliminary subgroup analysis was conducted. Two subsets were considered: (1) a sex-based comparison between five male and five female participants of comparable age; and (2) a lifestyle-based comparison between five physically active individuals and five sedentary ones. Boxplots in Fig. 13 illustrate the $RI_{\mathrm {PAT}}$ trajectories for each subgroup.

No formal statistical analysis was performed due to the limited sample size. However, in the sex-based comparison, males exhibited a faster recovery pattern than females. In the fitness-based comparison, the difference was more pronounced, with physically active subjects showing a faster trend back to baseline compared to sedentary ones.

## IV. Discussion

This work presented a clinically motivated proof-of-concept validation of a prototype wearable platform designed for synchronized acquisition of multiple physiological signals in a fully wireless multi-node configuration. Through a cuff-less blood pressure monitoring application based on pulse arrival time, the system was evaluated in a realistic use case with translational relevance for remote cardiovascular assessment. The aim was to verify that the proposed architecture can serve as an enabling engineering infrastructure for unobtrusive longitudinal monitoring of cardiovascular dynamics outside conventional clinical environments. In this perspective, the platform may support future workflows in which synchronized multi-signal tracking complements intermittent standard measurements, helping clinicians identify trends, recovery abnormalities, or context-dependent hemodynamic variations that would otherwise remain unobserved.

The analysis compared the proposed prototype with a reference sphygmomanometer and covered population-level dynamics, recovery modeling, and inter-subject variability, highlighting the platform as a promising translational infrastructure for future wearable and personalized digital health applications.

### A. Platform Architecture and Signal Processing Pipeline

The proposed platform relies on a dual-protocol architecture that handles synchronization and data transmission separately. A lightweight 2.4 GHz proprietary protocol maintains sub-microsecond synchronization across distributed nodes, while BLE manages data transfer to the external gateway. In practice, decoupling temporal alignment from data flushing is particularly valuable to preserve measurement integrity while the communication layer can be adapted to environmental and operational constraints (e.g., gateway placement, transmission scheduling, or bandwidth availability). This separation enables deterministic sampling even under non-ideal network conditions, and the continuous alignment between ECG and PPG channels provides the temporal precision required to preserve beat-by-beat PAT estimation despite sensors’ clock drift.

Signal processing was implemented via a fully automated pipeline. ECG and PPG were notch filtered at 50 Hz and then bandpass filtered (ECG: 0.5-40 Hz, PPG: 0.5-10 Hz). R-peaks were detected using a Pan-Tompkins algorithm, and PPG fiducial points ($PPG_{Md}$) were defined as maximal slope locations. PAT values ($PAT_{Md}$) were computed as the R-to-$PPG_{Md}$ delay, and a median value was extracted for each 10-second acquisition window. To mitigate the impact of motion artifacts and occasional low-quality segments, a conservative rolling-window outlier rejection was applied as a quality-control step. This step reflects the well-known susceptibility of wearable acquisitions to transient artifacts, especially with the use of low-cost analog commercial modules, where optical coupling, skin perfusion, sweat, and unstable sensor placement can degrade collected signals.

### B. PAT Dynamics and Cardiovascular Analysis

The platform was evaluated on 19 healthy adults (12 males, 7 females; aged 23-57). Aggregated results showed that PAT and SBP changed significantly after exercise, whereas DBP remained largely stable. Immediately post-exercise, PAT decreased in all subjects, reflecting increased pulse wave velocity, while SBP increased concurrently. Both variables then progressively returned toward baseline during recovery, producing the characteristic V-shaped PAT trend and its mirrored SBP pattern (Figs. 8–9). The adopted perturbation was intentionally modest and representative of a daily-life home monitoring scenario.

Data were further segmented into REST, EXERCISE, and RECOVERY phases. Due to normality violations, non-parametric testing was adopted. Kruskal-Wallis tests confirmed highly significant differences across phases for PAT and SBP ($p < 0.0001$), but not for DBP. Mann-Whitney post-hoc tests (Tables 4- 5) revealed significant PAT shortening from REST to EXERCISE ($p < 0.0001$) and significant lengthening from EXERCISE to RECOVERY ($p < 0.0001$), with no significant difference between REST and RECOVERY ($p = 0.2142$). SBP exhibited the complementary pattern, again with significant REST-EXERCISE and EXERCISE-RECOVERY differences ($p < 0.0001$) and no significant REST-RECOVERY change. These results indicate that the proposed wireless architecture is sensitive to transient vascular states and can support the recognition of physiologically meaningful events and transitions, such as, in this experimental setup, the peak of physical effort and the subsequent recovery. From a clinical viewpoint, this reinforces the role of PAT as a practical surrogate marker when continuous gold-standard pressure measurements are not possible and highlights the platform capability for prompt identification of deviations from baseline behavior, which may trigger adaptive system responses, alerts, or higher-level decision logic, without the need of cuff-based measurements that require the user’s active participation.

To compare recovery trends described by PAT and SBP, normalized Recovery Indices (RI) were computed for both variables, mapping maximal perturbation to −100% and baseline to 0%. Sparse SBP samples were oversampled using the mono-exponential model to align with PAT’s temporal resolution (Fig. 11). Subject-wise correlation analysis yielded strong alignment between $RI_{\mathrm {PAT}}$ and $RI_{\mathrm {SBP}}$, with an average Pearson coefficient $r = 0.93$ and all subjects reporting $p < 0.05$ after excluding two moderate outliers (Table 7). Bland-Altman analysis showed a small mean bias (1.58%) but wide limits of agreement (LoA: $-34.36\% / 37.53\%$), indicating that inter-subject variability remains a relevant factor for general interpretation. However, the Concordance Correlation Coefficient remained high ($\rho _{c} = 0.885$), confirming strong overall agreement between the two recovery descriptors. Taken together, these results suggest that PAT can reliably mirror SBP trends at the population level, supporting the platform performance in continuous trend-focused monitoring, while highlighting the need for subject-specific calibration strategies and multi-modal context when moving toward personalized clinical interpretation. Finally, preliminary subgroup comparisons suggested faster cardiovascular recovery in physically active subjects and slightly faster recovery in males, although no formal tests were performed due to sample size.

### C. Limitations and Future Developments

The present study should be interpreted as an engineering proof of concept conducted on a limited population of healthy adults. Although the adopted protocol was sufficient to demonstrate statistically significant physiological responses and to validate the operational feasibility of the synchronized wireless architecture in a clinically relevant use case, the sample size does not support broad clinical generalization. In particular, the absence of hypertensive subjects, elderly fragile populations, and patients with cardiovascular disease prevents conclusions regarding diagnostic performance or usability in populations for whom cuff-less monitoring may be most clinically relevant. Future translational studies should therefore extend the validation to larger and more heterogeneous populations, including patients with altered vascular properties, impaired autonomic regulation, or chronic blood pressure abnormalities.

The hemodynamic perturbation was intentionally modest to emulate daily-life exertion; although it triggered statistically significant SBP changes and a clear V-shaped PAT response, validation under stronger and more diverse stimuli is required to assess generalizability across a broader range of cardiovascular states. Robustness to motion artifacts was not directly validated under continuous dynamic exercise, since, in the present protocol, synchronized ECG and PPG were not acquired during treadmill running itself, but during seated windows before and after the exercise. The study was designed to evaluate post-exercise cardiovascular dynamics under controlled conditions and the used low-cost prototype hardware was not suitable for acquisitions during active sport exercise. As a consequence, the present study does not yet quantify the performance of the system under stronger motion conditions typical of fully outdoor monitoring, but the selected analog front ends were validated on the sensitivity to skin coupling, sweating, and repositioning. Future work will address this aspect through dedicated protocols and through improved sensing hardware and attachment strategies, in order to gain acquisition capability during active physical exercise. Absolute cuff-less BP estimation was not pursued. The present findings support the capability of the proposed platform to monitor cardiovascular dynamics and recovery trends in response to modest physiological perturbations. The study focused on verifying PAT’s ability to track relative BP fluctuations and to represent different cardiovascular states using the proposed wireless platform. This interpretation is consistent with the proof-of-concept nature of the study and should not be extended to direct quantitative interchangeability with clinical blood pressure measurement standards. Upcoming work will explore lightweight calibration schemes and multi-modal feature integration (e.g., PAT with heart rate or respiratory features) to support absolute estimation. Although PAT tracked SBP recovery coherently at the population level, the observed inter-subject variability and the use of intermittent cuff-based References indicate that additional validation against continuous beat-to-beat pressure monitoring methods will be required before the system can be considered for quantitative clinical deployment. Indeed, the oscillometric reference measurements were intentionally intermittent rather than continuous. This choice was motivated by practical constraints related to repeated cuff inflations, user burden, and protocol compatibility with a realistic home-monitoring-oriented setup. Nevertheless, it limits the temporal resolution of the reference signal and precludes direct beat-to-beat comparison between PAT and blood pressure. In the present analysis, this limitation was mitigated by focusing on recovery trends and by aligning sparse SBP samples to the PAT temporal axis through model-based interpolation. Future validation studies should incorporate continuous reference pressure measurements, such as beat-to-beat non-invasive monitoring systems or invasive reference techniques where ethically and clinically justified. Adoption of PTT, less sensitive to PEP than PAT, represents a further promising extension but requires additional sensing nodes (e.g., SCG/BCG) or dual-site PPG, which are naturally supported by the platform’s modular architecture. Future developments will target on-node processing for on-edge analysis and real-time feedback, facilitating remote telemonitoring and reducing analysis delays. Integrating real-time processing and adaptive decision logic could allow the system to autonomously react to variations in patients’ cardiovascular conditions, supporting timely clinical intervention in home monitoring scenarios, and to monitor hemodynamic fluctuations in patients at risk during daily life, increasing information for clinicians. The same synchronization architecture may also support different applications, such as segmental pulse wave velocity estimation or PAT-based autonomic arousal detection in sleep studies, while maximizing the patient comfort and increasing adherence. Another relevant development direction concerns the integration of data-driven AI-based blood pressure estimation models. In its current form, the platform was used to extract and analyze a single timing marker, PAT, in order to validate the synchronized wearable architecture in a clinically meaningful use case. However, the same infrastructure can support richer multi-parametric feature sets, including morphology-based PPG descriptors, heart-rate-related metrics, respiratory modulation features, or additional synchronized sensing modalities. This makes the platform suitable for future machine-learning or AI-based approaches aimed at personalized blood pressure estimation, adaptive calibration, or context-aware hemodynamic classification. Such developments will require larger datasets and clinically diverse populations, but the proposed architecture already provides the synchronized signal quality and modularity needed to support them.

Overall, these extensions will forward the prototype evolution from a research project into a versatile technological tool for multi-purpose, personalized, and context-aware remote health assessment.

## V. Conclusion

In conclusion, this work presented the design and proof-of-concept validation of a wearable platform for synchronized acquisition of multiple physiological signals in a clinically relevant cardiovascular monitoring scenario. The system achieved sub-microsecond alignment across distributed sensing units, enabling accurate fusion of ECG and PPG recordings for pulse arrival time analysis under realistic operating conditions. PAT derived from synchronized signals successfully captured exercise-induced hemodynamic changes and tracked systolic blood pressure recovery trends, supporting the feasibility of using the proposed architecture for trend-oriented cardiovascular monitoring. The fully wireless and modular design was robust and flexible under the tested conditions and enables repeated and unobtrusive acquisitions compatible with remote and home-based monitoring workflows, representing a promising translational infrastructure for future wearable and personalized digital health applications, subject to further clinical and real-world validation.

## References

[1] A. Serrani and A. Aliverti, “Feasibility study of an embedded platform for biopotentials acquisition during sports activities,” in Proc. IEEE Int. Workshop Sport, Technol. Res. (STAR), Cavalese, Italy, Jul. 2022, pp. 13–18, doi: 10.1109/STAR53492.2022.9859660.

[2] A. Serrani and A. Aliverti, “Data quality assessment for the validation of synchronization performance in an innovative wireless multi-node monitoring platform,” in Proc. IEEE Int. Conf. Metrol. eXtended Reality, Artif. Intell. Neural Eng. (MetroXRAINE), Milano, Italy, Oct. 2023, pp. 74–79, doi: 10.1109/METROXRAINE58569.2023.10405719.

[3] A. Serrani and A. Aliverti, “Performance assessment for the validation of wireless communication engines in an innovative wearable monitoring platform,” Sensors, vol. 24, no. 9, p. 2782, Apr. 2024, doi: 10.3390/s24092782. 38732888 PMC11086153

[4] S. L.-O. Martin, “Weighing scale-based pulse transit time is a superior marker of blood pressure than conventional pulse arrival time,” Sci. Rep., vol. 6, no. 1, p. 39273, Dec. 2016, doi: 10.1038/srep39273. 27976741 PMC5157040

[5] H. Gesche, D. Grosskurth, G. Küchler, and A. Patzak, “Continuous blood pressure measurement by using the pulse transit time: Comparison to a cuff-based method,” Eur. J. Appl. Physiol., vol. 112, no. 1, pp. 309–315, Jan. 2012, doi: 10.1007/s00421-011-1983-3. 21556814

[6] F. Beutel, C. Van Hoof, X. Rottenberg, K. Reesink, and E. Hermeling, “Pulse arrival time segmentation into cardiac and vascular intervals – implications for pulse wave velocity and blood pressure estimation,” IEEE Trans. Biomed. Eng., vol. 68, no. 9, pp. 2810–2820, Sep. 2021, doi: 10.1109/TBME.2021.3055154. 33513094

[7] R. Mukkamala, “Toward ubiquitous blood pressure monitoring via pulse transit time: Theory and practice,” IEEE Trans. Biomed. Eng., vol. 62, no. 8, pp. 1879–1901, Aug. 2015, doi: 10.1109/TBME.2015.2441951. 26057530 PMC4515215

[8] R. Mukkamala, G. S. Stergiou, and A. P. Avolio, “Cuffless blood pressure measurement,” Annu. Rev. Biomed. Eng., vol. 24, no. 1, pp. 203–230, Jun. 2022, doi: 10.1146/annurev-bioeng-110220-014644. 35363536

[9] R. Mukkamala, S. G. Shroff, K. G. Kyriakoulis, A. P. Avolio, and G. S. Stergiou, “Cuffless blood pressure measurement: Where do we actually stand?,” Hypertension, vol. 82, no. 6, pp. 957–970, Jun. 2025, doi: 10.1161/hypertensionaha.125.24822. 40231350 PMC12331212

[10] G. S. Stergiou, “Cuffless blood pressure measuring devices: Review and statement by the European society of hypertension working group on blood pressure monitoring and cardiovascular variability,” J. Hypertension, vol. 40, no. 8, pp. 1449–1460, Aug. 2022, doi: 10.1097/hjh.0000000000003224. 35708294

[11] J. Lee, S. Yang, S. Lee, and H. C. Kim, “Analysis of pulse arrival time as an indicator of blood pressure in a large surgical biosignal database: Recommendations for developing ubiquitous blood pressure monitoring methods,” J. Clin. Med., vol. 8, no. 11, p. 1773, Oct. 2019, doi: 10.3390/jcm8111773. 31653002 PMC6912522

[12] E. Finnegan, “Pulse arrival time as a surrogate of blood pressure,” Sci. Rep., vol. 11, no. 1, p. 22767, Nov. 2021, doi: 10.1038/s41598-021-01358-4. 34815419 PMC8611024

[13] Q. Zhang, D. Zhou, and X. Zeng, “Highly wearable cuff-less blood pressure and heart rate monitoring with single-arm electrocardiogram and photoplethysmogram signals,” Biomed. Eng. OnLine, vol. 16, no. 1, p. 23, Feb. 2017, doi: 10.1186/s12938-017-0317-z. 28166774 PMC5294811

[14] V. G. Ganti, A. M. Carek, B. N. Nevius, J. A. Heller, M. Etemadi, and O. T. Inan, “Wearable cuff-less blood pressure estimation at home via pulse transit time,” IEEE J. Biomed. Health Informat., vol. 25, no. 6, pp. 1926–1937, Jun. 2021, doi: 10.1109/JBHI.2020.3021532. PMC8221527 32881697

[15] S. Heimark, “Blood pressure response and pulse arrival time during exercise testing in well-trained individuals,” Frontiers Physiol., vol. 13, Jul. 2022, Art. no. 863855, doi: 10.3389/fphys.2022.863855. PMC9309297 35899026

[16] A. M. Carek, J. Conant, A. Joshi, H. Kang, and O. T. Inan, “SeismoWatch: Wearable cuffless blood pressure monitoring using pulse transit time,” Proc. ACM Interact., Mobile, Wearable Ubiquitous Technol., vol. 1, no. 3, pp. 1–16, Sep. 2017, doi: 10.1145/3130905. PMC6292433 30556049

[17] R. Lazazzera, Y. Belhaj, and G. Carrault, “A new wearable device for blood pressure estimation using photoplethysmogram,” Sensors, vol. 19, no. 11, p. 2557, Jun. 2019, doi: 10.3390/s19112557. 31167514 PMC6603632

[18] R. C. Block, “Conventional pulse transit times as markers of blood pressure changes in humans,” Sci. Rep., vol. 10, no. 1, p. 16373, Oct. 2020, doi: 10.1038/s41598-020-73143-8. 33009445 PMC7532447

[19] J. Choi, Y. Kang, J. Park, Y. Joung, and C. Koo, “Development of real-time cuffless blood pressure measurement systems with ECG electrodes and a microphone using pulse transit time (PTT),” Sensors, vol. 23, no. 3, p. 1684, Feb. 2023, doi: 10.3390/s23031684. 36772724 PMC9920508

[20] H. J. Baek, K. K. Kim, J. S. Kim, B. Lee, and K. S. Park, “Enhancing the estimation of blood pressure using pulse arrival time and two confounding factors,” Physiological Meas., vol. 31, no. 2, pp. 145–157, Feb. 2010, doi: 10.1088/0967-3334/31/2/002. 20009186

[21] T. G. Pickering, “Recommendations for blood pressure measurement in humans and experimental animals: Part 1: Blood pressure measurement in humans: A statement for professionals from the subcommittee of professional and public education of the American Heart Association Council on High Blood Pressure Research,” Circulation, vol. 111, no. 5, pp. 697–716, Feb. 2005, doi: 10.1161/01.cir.0000154900.76284.f6. 15699287

[22] P. Muntner, “Measurement of blood pressure in humans: A scientific statement from the American Heart Association,” Hypertension, vol. 73, no. 5, pp. e35–e66, May 2019, doi: 10.1161/hyp.0000000000000087. 30827125 PMC11409525

[23] R. Bartels-Ferreira, “Can a first-order exponential decay model fit heart rate recovery after resistance exercise?,” Clin. Physiol. Funct. Imag., vol. 35, no. 2, pp. 98–103, Mar. 2015, doi: 10.1111/cpf.12132. 24494748

[24] G. L. Pierpont, D. R. Stolpman, and C. C. Gornick, “Heart rate recovery post-exercise as an index of parasympathetic activity,” J. Autonomic Nervous Syst., vol. 80, no. 3, pp. 169–174, May 2000, doi: 10.1016/s0165-1838(00)00090-4. 10785283

[25] S. Heimark, “Blood pressure altering method affects correlation with pulse arrival time,” Blood Pressure Monitor., vol. 27, no. 2, pp. 139–146, Apr. 2022, doi: 10.1097/mbp.0000000000000577. PMC8893131 34855653

[26] G. Zhang, M. Gao, D. Xu, N. B. Olivier, and R. Mukkamala, “Pulse arrival time is not an adequate surrogate for pulse transit time as a marker of blood pressure,” J. Appl. Physiol., vol. 111, no. 6, pp. 1681–1686, Dec. 2011, doi: 10.1152/japplphysiol.00980.2011. 21960657

[27] Electronic or Automated Sphygmomanometers, ANSI/AAMI SP10-1992/A1, The Association for the Advancement of Medical Instrumentation, New York, NY, USA, 1996.

